# β-Arrestin Regulation of Myosin Light Chain Phosphorylation Promotes AT1aR-mediated Cell Contraction and Migration

**DOI:** 10.1371/journal.pone.0080532

**Published:** 2013-11-08

**Authors:** Elie Simard, Jeffrey J. Kovacs, William E. Miller, Jihee Kim, Michel Grandbois, Robert J. Lefkowitz

**Affiliations:** 1 Département de Pharmacologie, Faculté de Médecine et des Sciences de la Santé de l’Université de Sherbrooke, Sherbrooke, Québec, Canada; 2 Institut de Pharmacologie de Sherbrooke, Faculté de Médecine et des Sciences de la Santé de l’Université de Sherbrooke, Sherbrooke, Québec, Canada; 3 Chaire de Recherche Canadienne en Nanopharmacologie et Microscopie à Force Atomique, Faculté de Médecine et des Sciences de la Santé de l’Université de Sherbrooke, Sherbrooke, Québec, Canada; 4 Department of Medicine, Duke University Medical Center, Durham, North Carolina, United States of America; 5 Department of Biochemistry, Duke University Medical Center, Durham, North Carolina, United States of America; 6 Howard Hughes Medical Institute, Duke University Medical Center, Durham, North Carolina, United States of America; 7 Department of Molecular Genetics, University of Cincinnati College of Medicine, Cincinnati, Ohio, United States of America; Loyola University Chicago, Stritch School of Medicine, United States of America

## Abstract

Over the last decade, it has been established that G-protein-coupled receptors (GPCRs) signal not only through canonical G-protein-mediated mechanisms, but also through the ubiquitous cellular scaffolds β-arrestin-1 and β-arrestin-2. Previous studies have implicated β-arrestins as regulators of actin reorganization in response to GPCR stimulation while also being required for membrane protrusion events that accompany cellular motility. One of the most critical events in the active movement of cells is the cyclic phosphorylation and activation of myosin light chain (MLC), which is required for cellular contraction and movement. We have identified the myosin light chain phosphatase Targeting Subunit (MYPT-1) as a binding partner of the β-arrestins and found that β-arrestins play a role in regulating the turnover of phosphorylated myosin light chain. In response to stimulation of the angiotensin Type 1a Receptor (AT1aR), MLC phosphorylation is induced quickly and potently. We have found that β-arrestin-2 facilitates dephosphorylation of MLC, while, in a reciprocal fashion, β-arrestin 1 limits dephosphorylation of MLC. Intriguingly, loss of either β-arrestin-1 or 2 blocks phospho-MLC turnover and causes a decrease in the contraction of cells as monitored by atomic force microscopy (AFM). Furthermore, by employing the β-arrestin biased ligand [Sar^1^,Ile^4^,Ile^8^]-Ang, we demonstrate that AT1aR-mediated cellular motility involves a β-arrestin dependent component. This suggests that the reciprocal regulation of MLC phosphorylation status by β-arrestins-1 and 2 causes turnover in the phosphorylation status of MLC that is required for cell contractility and subsequent chemotaxic motility.

## Introduction

Cellular contraction, governed by actin-myosin-mediated motor activity, is central to many physiological processes in both muscle and non-muscle cells, including cell division, adhesion, chemotaxis and cytokinesis [1]. Dynamic mechanical activity also regulates contractile events that mediate vascular permeability in vascular endothelial cells and governs blood pressure in vascular smooth muscle cells [2]. Regulation of the tensile state of cells is therefore critical for organ homeostasis. Indeed, abnormal contraction of vascular smooth muscle cells can lead to hypertension and complications such as cardiac hypertrophy and failure, renal dysfunction and cerebrovascular maladies [3]. 

Several G-protein-coupled receptors (GPCRs), such as the Angiotensin II type 1a receptor (AT1aR), are involved in the regulation of cellular contraction. The AT1aR agonist, Angiotensin II (Ang II), a potent vasoconstrictor, is well known to contribute to the development of cardiovascular diseases [4]. ATa1R is coupled mainly to the Gαq/11 G-proteins and signals primarily through the downstream effectors phospholipase C and protein kinase C [3]. Additionally, it has been shown AT1aR is also coupled to Gα12/13 which has the small G-protein Rho as an effector [5]. Upon activation of AT1aR, β-arrestins are also recruited to the receptor to desensitize G-protein-mediated signaling and to act as scaffolding proteins [6,7] that mediate several other signaling pathways [8]. Included in these β-arrestin-mediated signaling pathways are the mitogen-activated protein kinase (MAPK) [9] and Rho-A-GTPase cascades, which have been shown to regulate both actin cytoskeletal reorganization and cell migration [5]. Recent proteomic studies revealed that the β-arrestins interact with more than 35 cellular structural proteins including cytoskeletal components such as actin, and motor proteins such as myosin [8].

Myosins (both smooth muscle and non-muscle) are regulated by the phosphorylation of MLC [10]. Phosphorylation of myosin catalyzed either by Myosin Light Chain Kinase (MLCK) or by Rho-activated Kinase (ROCK), activates myosin by promoting transition to an extended conformation. This conformation allows myosin to bind actin and use ATP to generate a force of contraction. Conversely, dephosphorylation of MLC by Myosin Light Chain Phosphatase (MLCP) stabilizes an inhibited and bent conformation with low affinity for actin. It has been proposed that cycling of MLC between its phosphorylated and unphosphorylated states is required for cell motility and contraction [11]. Turnover of phosphorylated MLC is regulated, in part, by MLCP, which is a heterotrimeric protein composed of a phosphatase (PP1C), a myosin binding and regulatory domain (MYPT), and a domain (ML20) of unknown function [12]. Interestingly, ROCK can affect MLC phosphorylation by directly phosphorylating MLC [13] or by phosphorylating MYPT and consequently inhibiting MLCP activity.

Several lines of evidence suggest a role for β-arrestins in the regulation of cellular contraction. We have previously reported that β-arrestin-1 is required for RhoA/ROCK activation and stress fiber formation [5]. *Godin et al*. also reported recently the importance for β-arrestins-1 and 2 in the regulation of Rho-A-dependent membrane blebbing [14]. Additionally, we have previously shown that β-arrestin-2 is required for Ang II-induced chemotaxis in HEK 293 AT1aR cells (AT1aR-293) [15]. These studies suggest a role for angiotensin-dependent β-arrestin signaling in dynamic cytoskeletal reorganization. 

Previously, a proteomics study from our group found that MYPT-1 interacts with β-arrestin-2 upon AT1aR activation [8]. Here, we present a yeast-II hybrid screen revealing a direct interaction between β-arrestin-1 and MYPT-1. Based on our previous data, and the fact that little is known about the consequences of the β-arrestin/MYPT-1 interaction, we set out to delineate the respective roles of β-arrestin-1 and 2 in regulating MLC phosphorylation by investigating the downstream consequences of this signaling interplay. Ours results confirm a key role for β-arrestin-mediated signaling in regulating cell contraction and motility. 

## Materials and Methods

### Reagents

Angiotensin II was purchased from Sigma. [Sar^1^, Ile^4^, Ile^8^]-Angiotensin II (SII) was purchased from the Cleveland Clinic Core Synthesis Facility. Q-serum was prepared as follows: 5ml Source 30Q beads (GE Healthcare Life Science) were washed two times in phosphate buffered saline (PBS) then incubated with 25 ml fetal bovine serum (FBS) (Sigma) for 30 min with agitation. The mixture was then centrifuged at 2000 rpm and the treated serum was recovered and filtered through 0.22 µm.

### Cell culture

AT1aR-stable HEK-293 (AT1aR-293) cells were maintained in minimum essential Eagle’s medium (EMEM) (M2279; Sigma) plus 10% FBS, 0.6 IU/ml penicillin (Sigma) and 600 µg/ml streptomycin (Sigma) in the presence of 100 μg/ml zeocin (Invitrogen) as described previously [16]. Rat vascular smooth muscle cells (rVSMC) were prepared from aortas of male Sprague-Dawley rats by enzymatic digestion [17] and maintained in Medium 199 (M199; Sigma) supplemented with 10% FBS, 0.6 IU/ml penicillin and 600µg/ml streptomycin. Slow growing early passage (less than 5) cells at 80-90% confluence were used for all experiments.

### Yeast two-hybrid Screen

The full length cDNA of rat β-arrestin-1 was subcloned into the yeast expression vector pAS2-1 using EcoRI and BamHI restriction sites. Following amplification in *Escherichia coli*, the pAS2-1 β-arrestin-1 plasmid was transformed into yeast strain PJ69-4A and a yeast two-hybrid screen was carried out essentially according to the Clontech Matchmaker protocol using a GAL4 activation domain fusion library in pACT2 (Matchmaker human heart cDNA library, Clontech). Library clones containing putative β-arrestin-1 interacting proteins were identified by positive growth on Trp/Leu/His/Ade dropout plates. Plasmids were isolated from positive clones using standard methodologies and analyzed by DNA sequencing (Howard Hughes Nucleic Acid Facility, Duke University). One of these clones corresponded to amino acids 643 to 943 of human MYPT-1 (NM_001143886). To confirm this interaction and to map the β-arrestin-1 interacting domain, DNA fragments corresponding to amino acids 1-163 or 1-253 of rat β-arrestin-1 were PCR amplified and subcloned into pAS2-1 using EcoRI and BamH1 restriction sites. Plasmids corresponding to full length (1-418), 1-163, or 1-253 β-arrestin-1 were then co-transformed with pACT2 MYPT-1(643-943) into the PJ69-4A yeast strain and selected on Trp/Leu dropout plates. Yeasts containing the β-arrestin-1 and MYPT-1 plasmids were then analyzed for growth on Trp/Leu/Ade dropout plates.

### Plasmid Transfection and siRNA

3 days before β-arrestin overexpression experiments, AT1aR-293 cells were transfected with either 1μg of plasmid pcDNA3-β-arrestin-1-FLAG or pcDNA3- β-arrestin-2-FLAG (encoding rat β-arrestins) [18,19] using FuGENE 6 (Roche Applied Science) transfection reagent according to the manufacturer’s instructions. 

3 days before β-arrestin and MYPT-1 siRNA-mediated knockdown experiments, 50% confluent human AT1aR-293 or rVSMC cells were transfected with siRNA using Gene Silencer (Gene Therapy Systems) as previously described [16,19] or Protein-Transduction Domain-Double-stranded RNA Binding-Domain (PTD-DRBD) reagent (a gift from Steven Dowdy’s lab, see protocol below) [20]. Unlabeled and YFP-tagged custom made control, human β-arrestin-1 and human β-arrestin-2 siRNAs were 5-uucuccgaacgugucacgudTdT-3, 5-agccuucugcgcggagaaudTdT-3 and 5-ccaaccucauugaauuugadTdT-3, respectively. The SMARTpool siRNA targeting human MYPT-1 consists of 4 different siRNA sequences (L-011340, Dharmacon). The custom made siRNA sequences targeting rat β-arrestin-1 and rat β-arrestin-2 were 5-AGC CUU CUG UGC UGA GAA C-3 and 5-CCA ACC TCA TTG AAT TCG A-3 respectively [21]. The SMARTpool siRNA targeting rat MYPT-1 consist of 4 different siRNA (L-095860, Dharmacon). Protein knockdown efficacy was determined by immunoblotting and calculated by comparing the levels of the target protein in cells transfected with the specified siRNA to the cells treated with the control siRNA. Data are expressed as % of reduction in protein expression. 

### PTD-dRBD siRNA delivery protocol

rVSMCs were seeded to 80% confluency in 6-well plates and cultured overnight. The following day, siRNA delivery system was prepared by mixing 10 µl of 40 µM siRNA diluted in water with 9 µl of 200 µM PTD-dRBD protein pre-diluted in 64.2 µl of PBS-10% Glycerol. While the siRNA delivery mix was incubating on ice for 15 minutes, cells were washed with serum free medium and incubated in 1 ml 10% Q-serum-medium. After incubation on ice, the PTD-dRBD:siRNA mix was diluted 1:5 in 10% Q-serum-medium. Media was removed from cells and replaced with 400 ul PTD-dRBD:siRNA complexes. After 4-6 hrs of incubation at 37°C in 5% CO2 incubator, the cells were washed twice with 10% FBS medium and kept under culture conditions until assays (motility or western blotting) were performed 96 hours later.

### Immunoprecipitation and immunoblotting

Cells were solubilized in lysis buffer containing 50 mM HEPES (pH 7.5), 0.5% Nonidet P-40, 250 mM NaCl, 2 mM EDTA, 10% (v/v) glycerol, 1 mM sodium orthovanadate, 1 mM sodium fluoride, 1 mM phenylmethylsulfonyl fluoride, 5 μg/ml leupeptin, 5 μg/ml aprotinin, 1 μg/ml pepstatin A and 100 μM benzaminidine. For immunoprecipitation, 15 μl of protein A/G plus-agarose (SantaCruz) and 1 μg of specified antibody were added to whole-cell lysates. After 3 hour incubation at 4 °C on a rocker platform, the immunoprecipitates were collected by centrifugation at 2,500 rpm for 2 min. The supernatant was discarded, whereupon the pellet was washed 4 times and then dissolved in sample buffer. For direct detection of protein levels in lysates, whole-cell lysates were directly used for SDS-polyacrylamide gel electrophoresis. SDS-PAGE were conducted on 10% polyacrylamide tris-glycine gels (Invitrogen) and transferred to nitrocellulose membranes for immunoblotting according to standard protocols. Immunoblots were performed using the following antibodies: β-arrestin-1 polyclonal A1CT and β-arrestin-2 polyclonal A2CT [22], FLAG M2 (F1804; Sigma), MYPT-1 (2634, Cell Signaling), phosphorylated-MLC (3671, Cell Signaling), Actin (A1978, Sigma) and Tubulin (T5293, Sigma). Chemiluminescent detection was performed with horseradish peroxidase-coupled secondary antibodies (Amersham Biosciences) and SuperSignal West Pico reagent (Pierce). Chemiluminescence was quantified by a charge-coupled device camera (Syngene ChemiGenius2); representative images are shown as inverted grayscale. Relative quantification of protein levels was performed by dividing the intensity of the band corresponding to the protein of interest by the intensity of the band mentioned as the loading control protein. 

### AFM Measurements

A custom-made AFM-based force measurement device was mounted on an inverted epifluorescent microscope (Carl Zeiss) as described previously [23]. Silicon nitride 0.01N/m cantilevers (HYDRA2R-100NG, AppNano) were used as force sensor for real-time monitoring of the cell contractile responses. Cells starved overnight in CO_2_-independent EMEM media containing HEPES at 37°c were adapted to room temperature one hour prior to an AFM experiment. YFP fluorescence was used to select cells that incorporated siRNAs and contrast phase microscopy was used to manually position the cantilever tip over the apical region of the cell. With the help of a 15 µM piezo scanner (Physik Instumente) an individual cell was approached until a contact force of approximately 200pN was measured. Baseline measurements were typically recorded for 5 mins after which vehicle injection was performed as control. After an additional 5 min, agonists were injected. Simultaneously to AFM data recording, micrographs were taken every second in phase contrast microscopy using a 40X objective and an AxioCam MRm camera (Carl Zeiss). All AFM experiments were performed at room temperature to limit mechanical vibration and thermal drift introduced by a heating/cooling system. Cell mechanical responses are reported as variations in cell height measured by the cantilever deflection (nm) and cell contractions were reported as the maximal variation of the cell height with respect to the baseline. 

### Chemotaxis Assays

Boyden-Chamber assays were performed in transwell chambers of 24-well inserts with 8-μm pore membranes coated with fibronectin (5µg/ml) for 1 hour. rVSMC were serum-starved for 2 hours in M199 then assays were conducted in serum-free M199 media as well. Agonists were placed in the lower chamber, and 100 000 cells were placed in the upper chamber and incubated for 5 h at 37°C and 5% CO_2_. Cells were then removed from the upper chamber and the upper side of the membrane, leaving only those cells that migrated through the membrane and to the lower chamber. Membranes were then fixed with 4% paraformaldehyde and stained with 1% crystal violet before being dried, excised, and mounted on microscope slides. Membrane densities were quantified using the GeneTools image analysis software (Syngene). The chemotactic index was calculated by dividing values from membranes in the stimulated conditions by values from control membranes (Vehicle).

QCM chemotaxis cell migration assays (ECM510, Millipore) were performed in 96-well plates. Briefly, rVSMC were starved overnight in M199 and 25 000 cells per well were used using the protocol supplied by the manufacturer. The cell fluorescence was measured by a fluorescence plate reader using 480/520 nm filter set. The chemotactic index was calculated by dividing values from the different treatments by values from control conditions.

## Results

**Figure 1:**
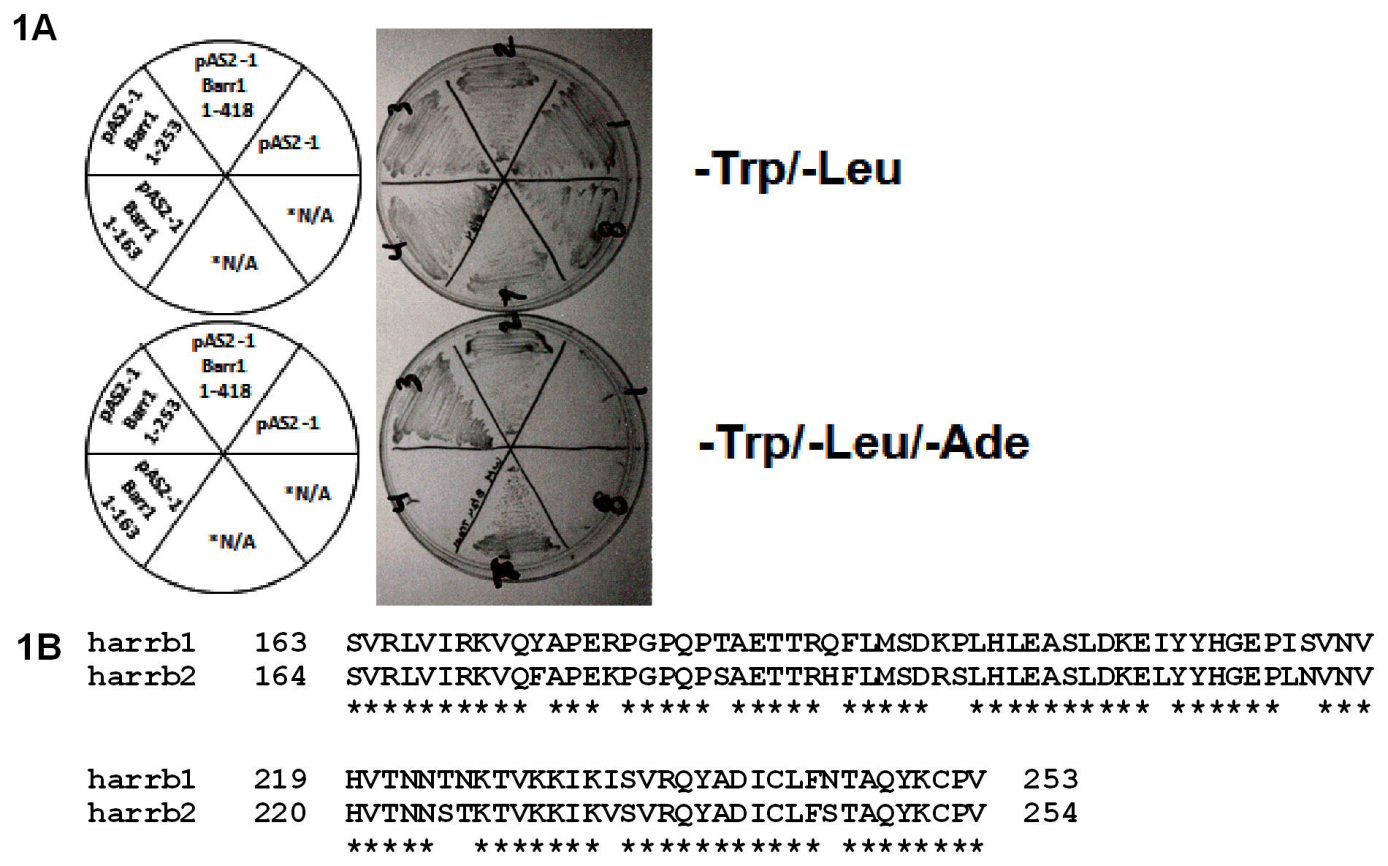
β-Arrestin-1 interacts with MYPT-1. **A**, β-Arrestin-1 constructs comprising amino acids 1-418 (full-length), 1-253 or 1-163 were co-transformed with a MYPT-1 construct containing amino acids 643-693 (the regulatory and myosin binding domains of MLCP). 1-418 and 1-253 β-arrestin-1 constructs interacted with MYPT-1 as indicated by growth on Ade dropout plates, while the 1-163 β-arrestin-1 construct or empty vector (pAS2-1) did not. Therefore, it appears that amino acids 163-253 of β-arrestin-1 contain the MYPT-1 interacting domain. * N/A sectors are unrelated experiments. **B**, β-Arrestin-1 (163-253) domain alignment with the corresponding β-arrestin-2 domain. * Indicates conserved amino acids between β-arrestin-1 and 2.

 In order to identify binding partners of β-arrestin-1, a yeast-II hybrid screen was performed using pAS2-1β-arrestin-1 as the bait and a human heart cDNA library as the prey. We isolated one clone corresponding to the c-terminus amino acids (643 to 943) of MYPT-1 (NM_001143886). Amino acids 643 to 943 contain the regulatory and myosin binding domains of the MLCP complex, indicating that these domains confer the ability of MLCP to interact with β-arrestin-1. To determine the regions of β-arrestin-1 that interact with MYPT-1, β-arrestin-1 fragments were co-expressed with MYPT-1(643-943) in a yeast-II hybrid assay. The results demonstrate that β-arrestin-1 (1-418) and (1-253) interact with MYPT-1(643-943), while β-arrestin-1 (1-163) did not (Figure **1A**). These results suggest that amino acids (163-253) of β-arrestin-1 contain the MYPT-1 binding domain. Intriguingly, proteomic experiments have shown that β-arrestin-2 also interacts with MYPT-1 in an AT1aR stimulus dependent fashion, suggesting that both β-arrestins bind MYPT-1 [8]. Worthy of notice, the amino acid sequence of β-arrestin-1 and β-arrestin-2 is highly similar in this region (Figure **1B**).

**Figure 2:**
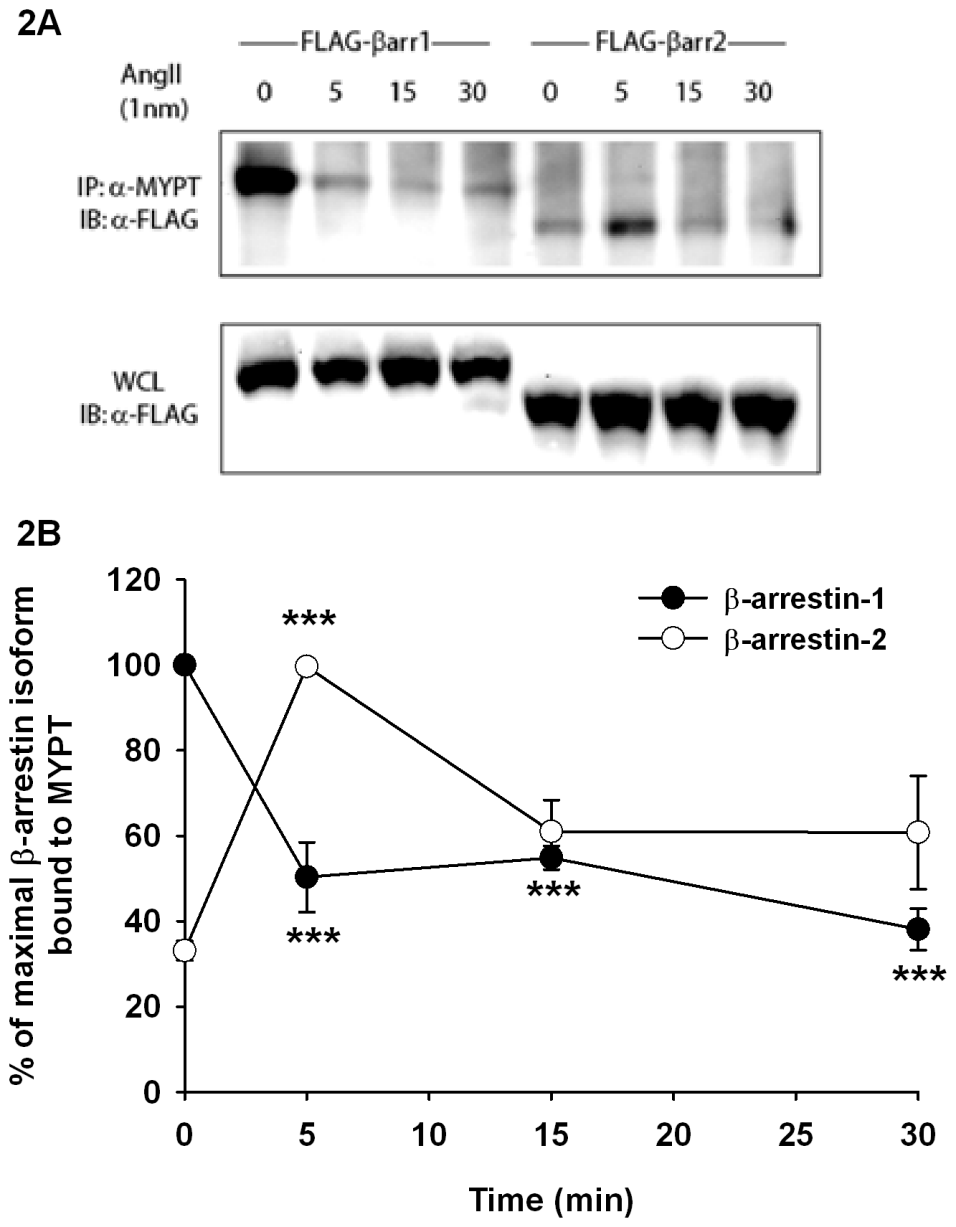
β-Arrestin-1 and 2 interact with MYPT-1. **A**, AT1aR-293 cells were transfected to express either β**-**arrestin-1-FLAG or β**-**arrestin-2-FLAG then stimulated with Ang II 1 nM for the indicated time. The interaction between overexpressed β**-**arrestin and endogenous MYPT-1 was analyzed by immunoprecipitation of MYPT-1 and revelation of β**-**arrestin by immunoblotting the FLAG tag. **B**, Semi-quantitative analysis of β-arrestin and MYPT interaction (n=4). For each experiment, the β**-**arrestin band with the highest intensity was assigned to 100% and all other bands were normalized as a percentage of that value. Data are presented as mean±standard mean error where *** *p*<0.001 compared with respective unstimulated condition (0 min) using one-way ANOVA with Bonferroni correction.

 To confirm the interaction of β-arrestins with MYPT-1, co-immunoprecipitations were performed following Ang II (1 nM) stimulation of AT1aR-293 cells overexpressing β-arrestin-1 or β-arrestin-2. Figure **2** shows that β-arrestin-1 interacts with MYPT-1 in resting cells, an interaction which diminishes rapidly following Ang II stimulation. On the other hand, β-arrestin-2 weakly interacts with MYPT-1 in basal condition. However, in presence of Ang II, this interaction is transiently increased to reach a maximum within 5 minutes. Taken together, those results confirm that β-arrestin-1 and 2 interact with MYPT-1 and suggest they may play a role in the regulation of the phosphorylation of MLC. 

**Figure 3:**
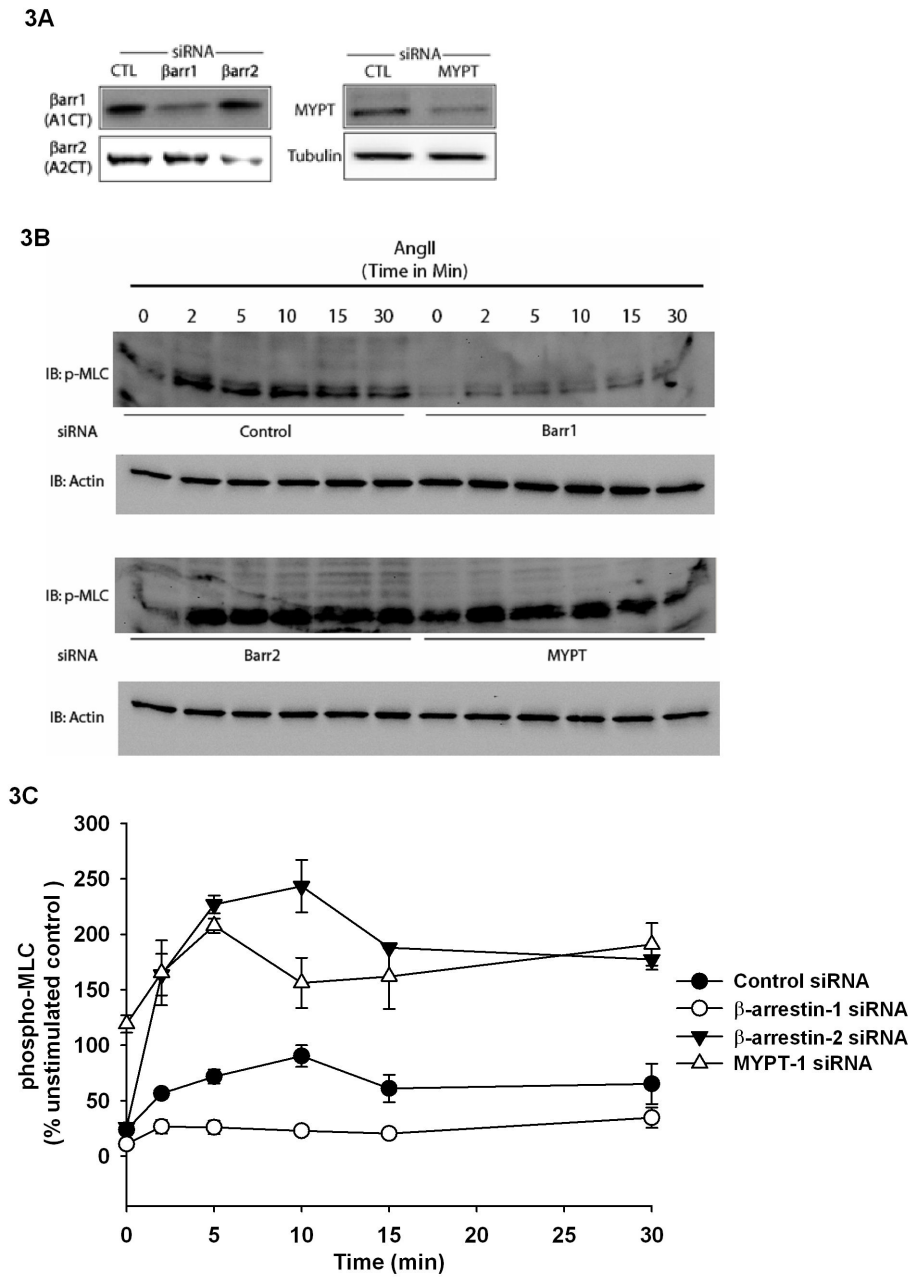
β-Arrestin-1 and β-arrestin 2 have opposing role on MLC phosphorylation. **A**, AT1aR-293 cells were transfected with siRNAs towards indicated proteins and immunoblotting was used to determine knockdown efficacies for indicated targets. **B**, AT1aR-293 cells were stimulated with Ang II 1nM for the indicated time after knockdown with indicated siRNAs. Phospho-MLC levels were analyzed by immunoblotting where actin was used as a loading control. **C**, Semi-quantitative analysis of phospho-MLC levels (n=4), where phospho-MLC levels are normalized according to respective actin levels then expressed as % of unstimulated control siRNA (0 min). Data are presented as mean±standard mean error where *p*<0.01 (5 min) and *p*<0.05 (10 min) for cells depleted in β-arrestin-1; *p*<0.01 (2, 10, 15 and 30 min) and *p*<0.001 (5 min) for cells depleted in β-arrestin-2 and *p*<0.05 (10 min) , *p*<0.01 (2 and 15 min) and *p*<0.001 (0, 5 and 30 min) in cells depleted in MYPT-1 compared to Control siRNA sample at given time points using one-way ANOVA with Bonferroni correction.

Work from our group and others has revealed a critical role for both β-arrestins in cytoskeletal dynamics downstream of the AT1aR [5,15]. Since both β-arrestins bind MYPT-1, it suggests that this interaction may affect the levels of phosphorylated myosin light chain in cells. To investigate the roles of β-arrestin-1 and 2 in regulating MLC phosphorylation, we acutely knocked down β-arrestins expression by transiently transfecting AT1aR-293 cells with siRNAs targeting β-arrestin-1, β-arrestin-2 or MYPT-1. Figure **3A** confirms that the knockdown of the different proteins of interest was effective. For all AT1aR 293 cells experiments, efficacies were evaluated and found to be 85.3±3.8 % for β-arrestin-1, 78.6±5.4% for β-arrestin-2 and 88.7±6.9% for MYPT-1. Control and knocked down cells for β-arrestin-1, β-arrestin-2 or MYPT-1 were then analyzed for MLC phosphorylation in response to Ang II (1nM). Stimulation with Ang II rapidly induces MLC phosphorylation in control cells reaching a maximum between 2 and 10 minutes (Figure **3B** **, upper left and 3C, solid circles**) and then showing a slight decrease before leveling off as the turnover of phospho-MLC reaches an apparent equilibrium for at least 30 min. In cells knocked down for β-arrestin-1, Ang II-stimulated MLC phosphorylation is essentially ablated (Figure **3B** **, upper right and 3C, open circles**) suggesting that β-arrestin1 is required for Ang II driven MLC phosphorylation. Interestingly, Ang II stimulation of β-arrestin-2 knockdown cells leads to a dramatic increase in MLC phosphorylation, (Figure **3B** **, lower left and 3C, filled triangles**), suggesting that β-arrestin-2 normally functions to suppress phosphorylation of MLC. MYPT-1 knockdown leads to a significant increase in both basal and Ang II stimulated MLC phosphorylation as would be predicted for cells lacking MLCP activity (Figure **3B** **, lower right and 3C, open triangles**). Since the loss of β-arrestin-2 or MYPT-1 leads to a similarly high level of MLC phosphorylation, our results suggest that β-arrestin-2 is a positive regulator of MYPT-1 and, consequently, MLCP activity. Taken together, these data point towards opposing roles for β-arrestin-1 and β-arrestin-2 on MLC phosphorylation where β-arrestin-1 promotes MLC phosphorylation while β-arrestin-2 acts as a repressor of MLC phosphorylation by promoting the activity of MYPT-1 and the MLCP complex. These experiments identify the β-arrestins as important regulators of MLC phosphorylation and suggest that they may in turn affect myosin-dependent cell contraction and motility. 

**Figure 4:**
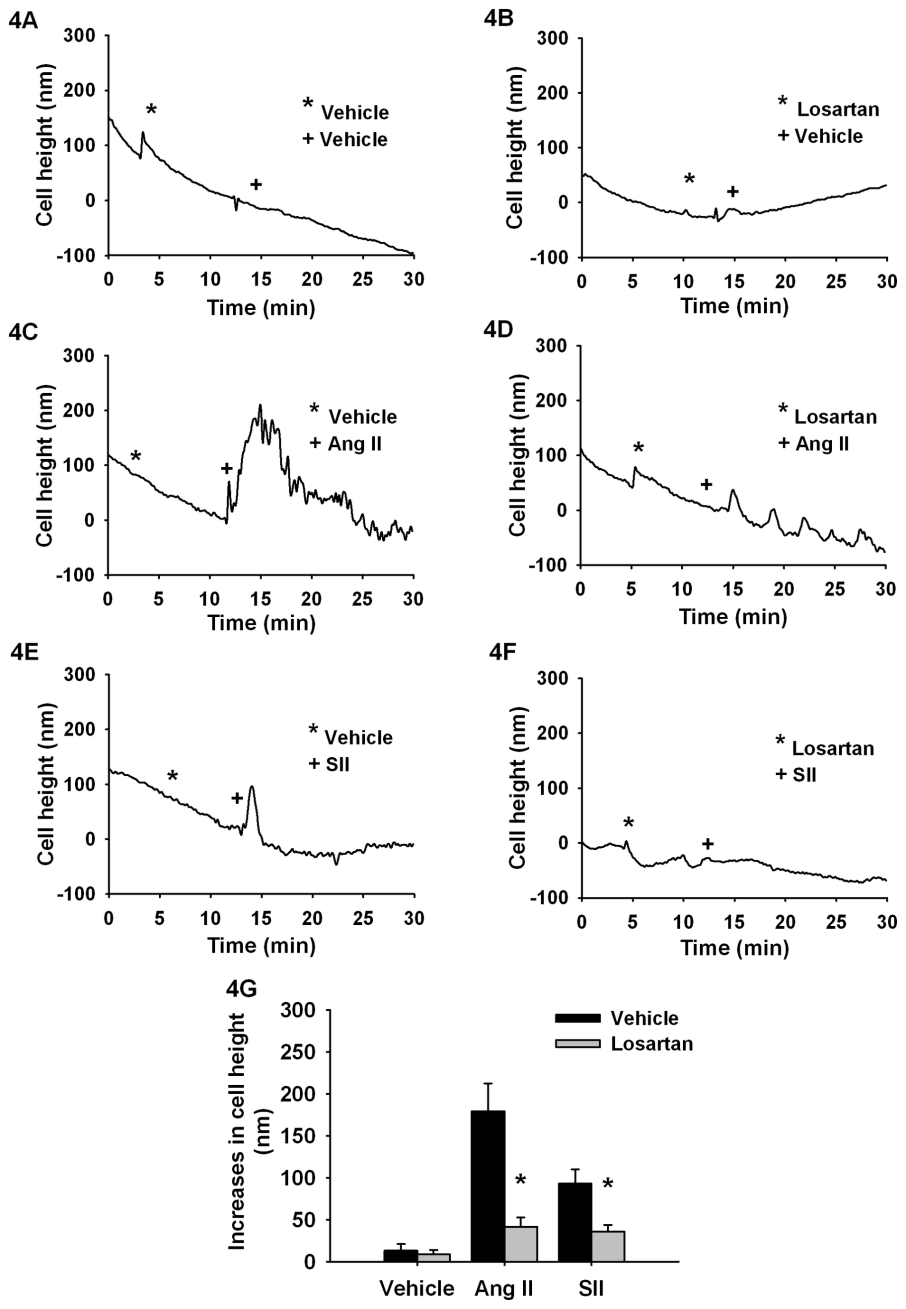
Ang II and SII induce cell contraction detectable by AFM. Typical AFM curves monitoring the cellular contraction following stimulations with (**A**) Vehicle, (**B**) Losartan, (**C**) Ang II, (**D**) Losartan and Ang II (**E**) SII (**F**) Losartan and SII. **G**, Histogram showing average cell contraction (maximal cell height variation) of cells pretreated with Vehicle or Losartan 100 nM and stimulated with Ang II 1 nM or SII 10 µM. Each bar represents 10-12 independent experiments and EMEM was used as vehicle. Data are expressed as mean±standard mean error where * *p*<0.05 compared with respective Ang II and SII responses using Student *t*-test.

Since phosphorylation of MLC is associated with cell contractility and motility, we next determined if these β-arrestin-mediated regulations of MLC phosphorylation could also affect cellular contraction. In order to examine this question, we conducted AFM-based measurements of single cell contractility in response to AT1aR activation triggered by the full agonist Ang II (1nM) or the β-arrestin biased agonist SII (10 µM) (Figure **4**). AFM experiments quantify the contraction of the cell body as a rapid increase in cell height following the stimulation with agonists. Compiled independent experiments (Figures 4C and 4E), show that Ang II and SII elicit a contractile response of 179±33 nm and 93±17 nm, respectively. Those responses were also blocked by the AT1aR antagonist Losartan (Figures 4D and 4F), thus confirming the specificity of the contractile responses to the activation of AT1aR. Since Ang II elicits both G-protein-mediated and β-arrestin-mediated signalling responses, while the SII elicits only a β-arrestin-mediated signaling response, the smaller contractile response induced by SII suggests that there are two components to contractile events downstream of the AT1aR; one governed by G-protein-mediated signalling and one governed by β-arrestin signaling.

**Figure 5:**
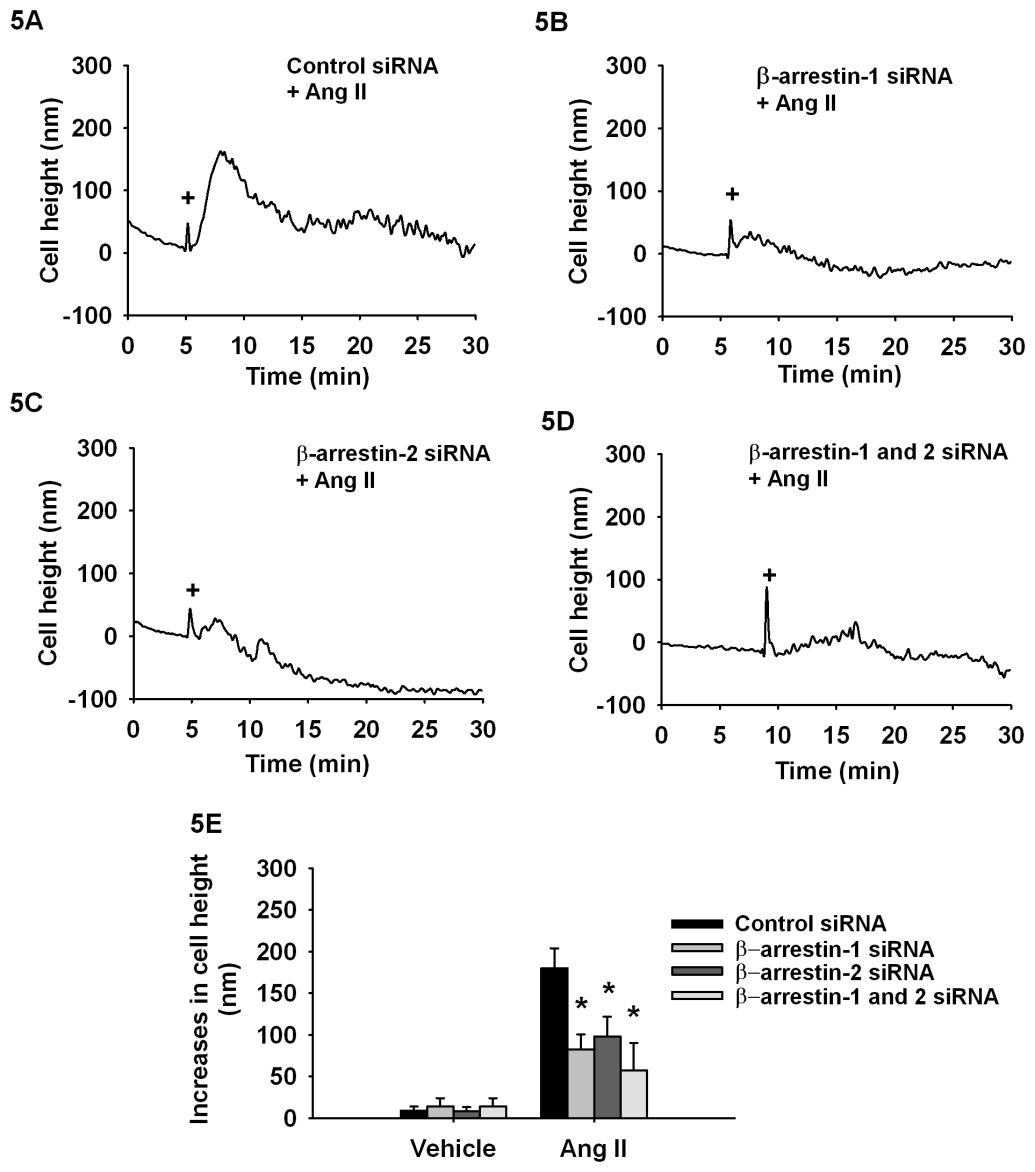
β-Arrestin 1 and 2 are participating in Ang II-dependent cell contraction. Typical AFM curves monitoring the cellular contraction following stimulations with (**A**) control, (**B**) β-arrestin-1, (**C**) β-arrestin-2 or (**D**) β-arrestin-1 and 2 siRNAs on Ang II-induced cellular contraction. **E**, Histogram showing average cell contraction (maximal cell height variation) (contraction) of cells transfected with indicated siRNA and stimulated with Ang II 1 nM. Each bar represents 10-12 independent experiments and EMEM was used as vehicle. Data are expressed as mean±standard mean error where * *p*<0.05 compared with control transfection response using one-way ANOVA followed by Dunnett post-test.

To examine the contribution of the β-arrestins in Ang II-dependent cell contraction, we conducted similar AFM experiments in cells transfected with β-arrestin-1 or β-arrestin-2 siRNA. When compared to the contractile response observed with Ang II (1nM) stimulation (179±24 nm), the loss of β-arrestin-1 or 2 leads to smaller contractions of 82±18 nm and 97±24 nm, respectively (Figures 5B and C). Moreover, the repression of both β-arrestin-1 and 2 (Figure **5D**) further decreases contractility (57±33 nm). This further supports the notion that cellular contraction induced by Ang II involves a G-protein-mediated component that is independent of β-arrestin signalling.

**Figure 6:**
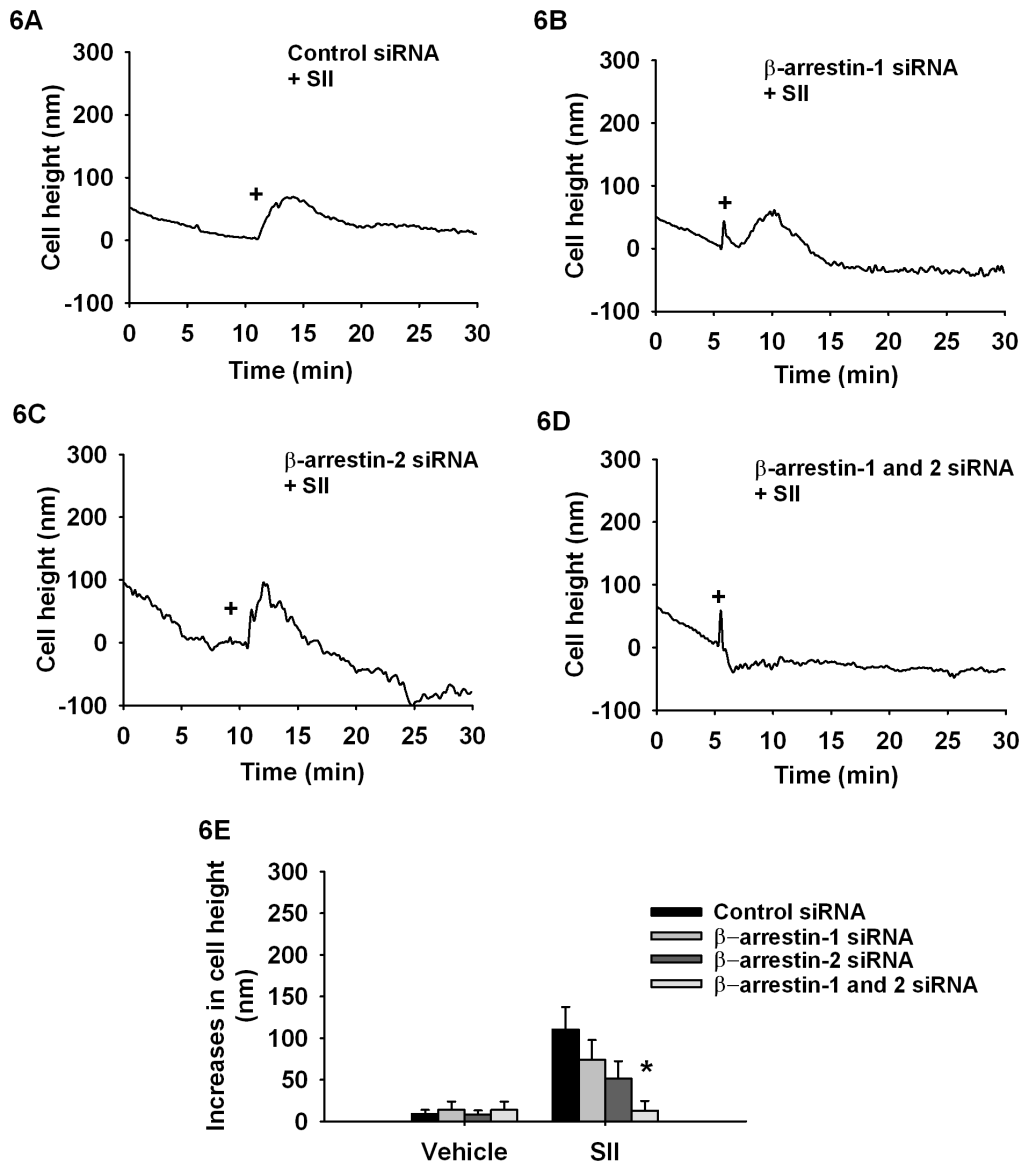
SII acts as a biased agonist inducing contraction via both β-arrestin-1 and 2 activation. Typical AFM curves monitoring the cellular contraction following stimulations with (**A**) control, (**B**) β-arrestin-1, (**C**) β-arrestin-2 or (**D**) β-arrestin-1 and 2 siRNAs on SII-induced cellular contraction. **E**, Histogram showing average cell contraction (maximal cell height variation) of cells transfected with indicated siRNA and stimulated with SII 10 µM. Each bar represents 10-12 independent experiments and EMEM was used as vehicle. Data are expressed as mean±standard mean error where * *p*<0.05 compared with control transfection response using one-way ANOVA followed by Dunnett post-test.

To determine if the SII-mediated effects on contractility seen in Figure **4** are the result of β-arrestin signaling and to identify the respective contributions of β-arrestin-1 and 2 in this response, we knocked down β-arrestin-1 or β-arrestin-2 expression prior to stimulation with SII (10µM) (Figure **6**). Interestingly, SII-dependent contractility (110±27 nm) was slightly decreased in cells lacking β-arrestin-1 (74±24 nm; Figure 6B and 6E) or β-arrestin-2 (51±21 nm; Figure 6C and 6E), whereas cells lacking both β-arrestins (13±12 nm) no longer responded (Figure 6D and 6E). These results confirm, through use of the β-arrestin biased ligand, SII, that β-arrestin-mediated signalling is actively involved in cell contraction. Moreover, these results support the hypothesis that β-arrestin-regulated phospho-MLC turnover is necessary for successful cytoskeletal reorganization and membrane contraction [11]. Indeed, in cells lacking one or both β-arrestins, these events are compromised and this correlates with changes in the magnitude and timing of MLC phosphorylation. 

**Figure 7:**
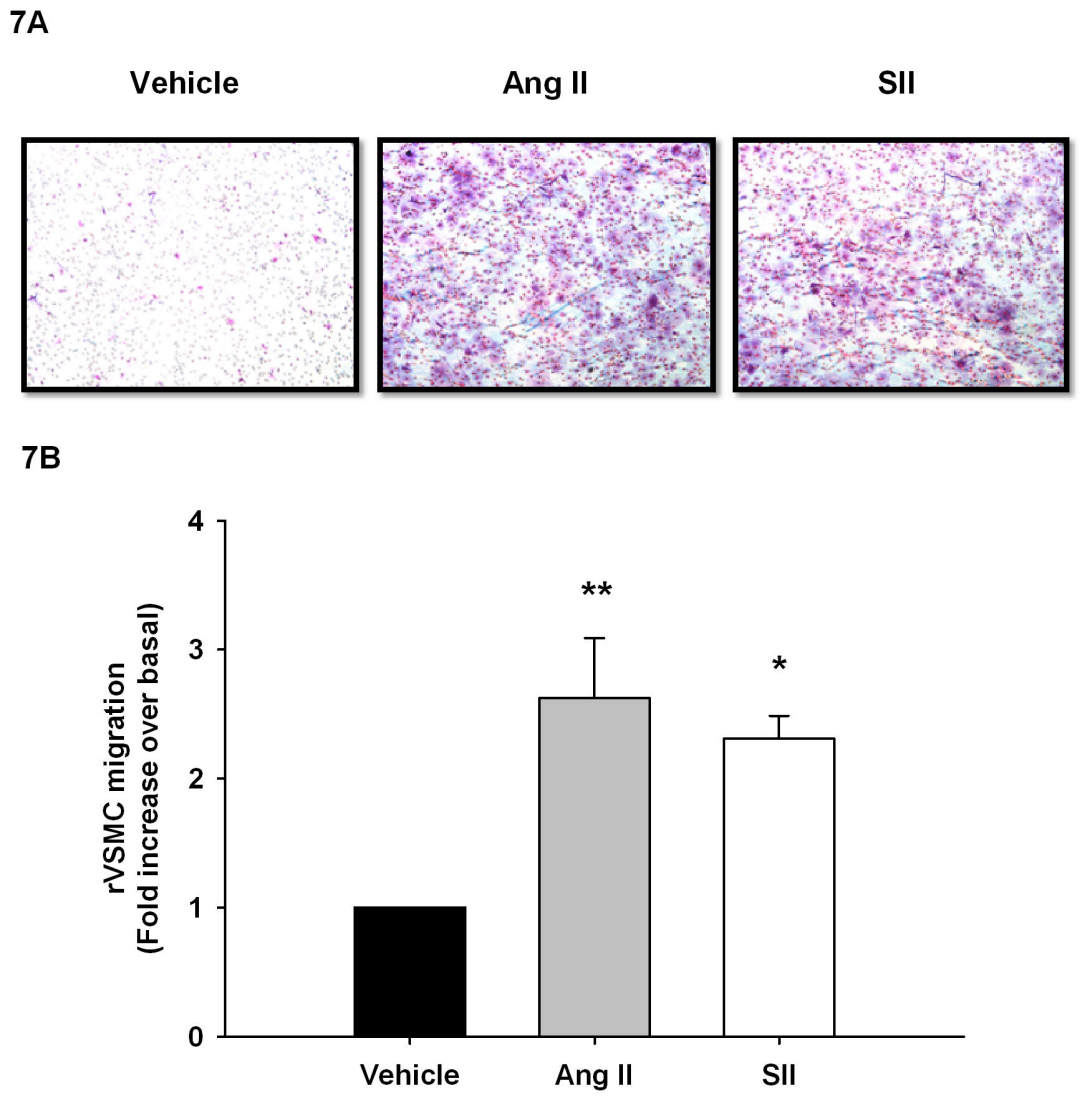
Ang II and SII induce rVSMC migration. **A**, Crystal violet staining of membranes following transwell migration assay of rVSMC exposed to Ang II 1 nM or SII 1 µM. **B**, Histogram showing Ang II and SII induced chemotaxis in rVSMC (n=5). Data are expressed as mean±standard mean error where * *p*<0.05 and ** *p*<0.01 compared with vehicle using one-way ANOVA with Bonferroni correction.

**Figure 8:**
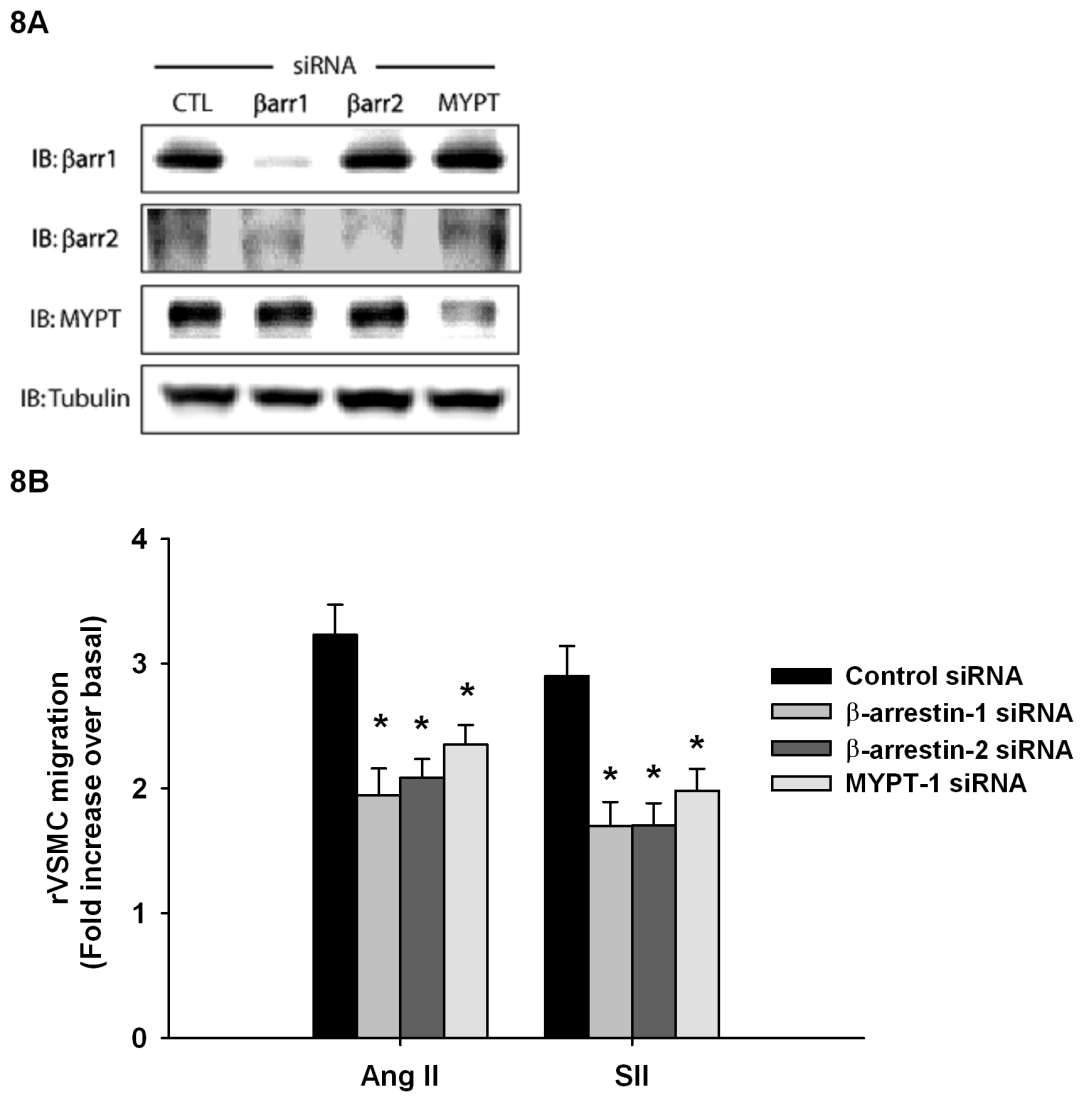
β-Arrestins are required for cell migration. **A**, rVSMC were transduced with siRNAs toward the indicated proteins. Immunoblots of representative rVSMC lysates from cell populations used in motility experiments were performed to confirm knockdown. **B**, rVSMC motility was examined using high-throughput QCM migration assay. Cell migration was assessed in response to Ang II 10 nM or SII 1 µM stimulation after 3 hours. Histogram showing Ang II and SII induced chemotaxis in conditions of β-Arrestin-1, 2 and MYPT-1 depletion. Data are expressed as mean±standard mean error where * *p*<0.05 compared with control transfection responses using one-way ANOVA with Bonferroni correction.

Since it appears that both β-arrestins play a role in the regulation of MLC phosphorylation, and both are required for AT1aR-induced cellular contractility, we wished to examine the involvement of β-arrestin-1, β-arrestin-2, and MYPT-1 in a physiologically relevant system that expresses endogenous levels of AT1aR receptors. Therefore, we used primary cultures of rat vascular smooth muscle cells (rVSMC) as they endogenously express the AT1aR and chemotax robustly in response to Ang II (1nM; 2.6±0.5 fold) or SII (1µM; 2.3±0.2 fold) (Figure **7**). The rVSMCs were then transfected with β-arrestin-1, β-arrestin-2, or MYPT-1 siRNAs and cell migration was assessed under Ang II or SII stimulation. Western blot analysis shows that knockdowns of β-arrestin-1, β-arrestin-2 or MYPT-1 in rVSMC were successful and did not affect the expression of other proteins of interest (Figure **8A**). Knockdown efficacies were evaluated from multiple independent experiments and found to be 92.4±10.2 % for β-arrestin-1, 86.4.6±7.1% for β-arrestin-2 and 76.3±9.3% for MYPT-1. Again, results show that chemotaxis is induced by both Ang II (10nM; 3.2±2 fold) and SII (1µM; 2.9±0.2 fold) in rVSMCs transfected with control siRNA. However, when the expression of β-arrestin-1 was repressed, the chemotactic response to either Ang II (1.9±0.2 fold) or SII (1.7±0.2 fold) was diminished (Figure **8B**). Similar results could be found in cells where β-arrestin-2 expression was inhibited and stimulated with Ang II (2.1±0.1 fold) or SII (1.7±0.2 fold) (Figure **8B**). Furthermore, results show that MYPT-1 removal also decreased rVSMC motility following Ang II (2.4±0.2 fold) or SII (2.0±0.2 fold) stimulation (Figure **8B**). These data confirm that β-arrestin-mediated signaling plays a major role in promoting cell contractility and chemotaxis downstream of the AT1aR. Furthermore, it seems that the reciprocal regulation of phosphorylated MLC by β-arrestins-1 and 2 is a part of this interplay.

## Discussion

The activation of distinct GPCRs has been reported to initiate numerous physiological responses including contraction in muscle and non-muscle cell types. In addition to their G-protein-mediated signaling, these receptors also trigger the recruitment and activation of β-arrestins that are not only responsible for mediating receptor internalization and desensitization, but also for driving G-protein-independent, β-arrestin-mediated signaling. The AT1aR is well-known to induce contractile responses in muscle cells [24]. Moreover, similar results have been found in non-muscle HEK-293 cells which overexpress AT1aR [23,25]; a well-established model system for elucidating AT1aR downstream signaling events. Interestingly, we have previously shown that β-arrestin-1 is required for Rho A activation and subsequent stress fiber formation and cytoskeleton rearrangement [5]. Furthermore, we have also shown that β-arrestin-2 is required for Ang-II-induced chemotaxis [15], thus implicating both β-arrestins in the contraction-motility signaling axis. Here we build upon those previous findings to further elucidate the mechanism whereby the β-arrestins regulate agonist-induced cell contraction through their interplay with MLC. 

We have shown here that the β-arrestin-mediated regulation of phosphorylated MLC is a significant component of AT1aR-induced mechanical response. Depletion of β-arrestin-1 from cells decreases levels of phospho-MLC in response to AT1aR stimulation (Figure **3**) suggesting a hyperactive MLCP complex. This regulatory event could be due to a simple sequestration of MYPT-1 away from the MLCP complex, or as suggested by our previous work [8] could be due to a loss of β-arrestin-1-dependent activation of Rho A. This would consequently lead to a loss of ROCK activation and hyperactive MLCP. Most likely, it is a combination of these factors that causes the effects seen by loss of β-arrestin-1. 

Additionally, we have shown that β-arrestin-2 is important for AT1aR-mediated dephosphorylation of MLC (Figure **3**). Agonist stimulation leads to higher levels of phospho-MLC in the absence of β-arrestin-2; suggesting that a β-arrestin-2/MYPT-1 complex may limit the access of MLCP to its substrate resulting in low activity of MLCP. Moreover, the loss of MYPT-1 in this system mimicked the loss of β-arrestin-2, thus lending further credence to the importance of MLCP activity and its reciprocal regulation by β-arrestins in phospho-MLC turnover. Further investigation will be needed to completely understand the spatial and temporal interplay of the β-arrestins, MYPT-1 and the MLCP complex in response to AT1aR stimulation. ERK 1/2 [9] and several other signaling pathways are also involved in β-arrestin signaling [8]. Interestingly, it has been reported that ERK can phosphorylate MLCK and participate, to a small extent, in myosin light chain phosphorylation, cell contraction and force generation [26]. However, to our knowledge, no link has been made between β-arrestin-dependent ERK activation and MLCK regulation. 

In 2008, Matsumura et al. proposed a model where high levels of phosphorylated MLC do not necessarily translate into increased cell contraction or migration. In their model, high MLC phosphorylation coupled with low MLCP activity result in low phospho-MLC turnover leading to increased cell adhesion and stress fiber formation. Conversely, high MLC phosphorylation coupled with high MLCP activity result in high phospho-MLC turnover leading to morphological changes and cell motility by accelerating focal adhesion turnover and limiting myosin-induced actin polymerization [11]. Our results suggest that the β-arrestins are required for this dynamic turnover of phospho-MLC. Moreover, in MYPT-1 depletion experiments, levels of phospho-MLC were found to be very high after Ang II treatment, and not surprisingly, MYPT-1 depletion in rVSMC reduced cell migration in response to both Ang II and SII. These results mirror those seen with loss of β-arrestin-2, and suggest that defective phospho-MLC turnover due to loss of β-arrestin-regulated MLCP activity leads to reduced cellular migration. 

Cycling of MLC phosphorylation correlates with cell contractility [27] and we showed that activation of AT1aR signaling through β-arrestins induces an increase in phosphor-MLC that can be correlated with cellular contraction events. We also observed that, SII, a β-arrestin-biased ligand for the AT1aR, can induce cellular contraction and cell migration (Figure **4**-**8**). Interestingly, while the two β-arrestins appear to reciprocally regulate phosphorylation of MLC, loss of either β-arrestin-1 or β-arrestin-2 function appears to have similar effects on cell contractility and motility. Considering that phosphorylation of MLC is a key event in cellular contraction and a driver of cellular migration, we believe that a failure to properly regulate MLC cycling in the absence of β-arrestin-mediated signalling is manifested as defective cellular contractility and migration.
